# Supplementary material for: Observational study of the clinical performance of a public-private partnership national referral hospital network in Lesotho: Do improvements last over time?
Source: PLoS One. 2022 Sep 28;17(9):e0272568. doi: 10.1371/journal.pone.0272568 (PMC9518856; doi:10.1371/journal.pone.0272568)
Supplement: S1 Table — (DOCX) [file pone.0272568.s002.docx]

| **2018 ward name** | **Ward description** |
| --- | --- |
| Short stay medical/surgical | Treats male and female patients requiring short stay medical/surgical procedures, including ophthalmologic procedures. The short stay medical/surgical ward did not exist in 2012 and was combined with the ophthalmology ward in 2018. |
| Orthopedic | Treats female and male patients requiring orthopedic procedures. |
| Female Medical | Treats female patients requiring medical but not surgical intervention. |
| Male Medical | Treats male patients requiring medical but not surgical intervention. |
| Female Surgical | Treats female patients requiring general surgical procedures. |
| Male Surgical | Treats male patients requiring general surgical procedures. |
| ICU | Treats the most critical patients of the hospital requiring high-dependency supportive care. |
| Gynecology | Treats female patients requiring gynecologic procedures. |
| Antenatal ^a^ | Treats female patients with antenatal complications and women in labor. |
| Postnatal ^a^ | Treats postpartum female patients immediately after labor and those with postnatal complications. |
| Nursery | Houses all healthy neonates born at QMMH. Only ill newborns requiring observation are formally counted as admissions. Healthy newborns are not counted as admissions. |
| Neonatal | Treats neonates requiring additional care and observation, including kangaroo care. Ward did not exist in 2012. |
| NICU | Treats neonates in the most critical condition requiring high-dependency supportive care. |
| Pediatric Medical | Treats female and male pediatric patients (aged 14 and under) requiring medical not surgical intervention. |
| Pediatric Surgical | Treats female and male pediatric patients (aged 14 and under) requiring surgical procedures. |
| Step Down  (Lodger) | Provides intermediate care. A patient may be admitted here first before needing further care in a medical or surgical ward or may transfer here after receiving treatment in another ward. Also houses women whose babies are in the neonatal or NICU wards to allow them to continue nursing and providing kangaroo care when appropriate. |
| Observation A&E | Treats and observes patients who were admitted through the A&E Department while they await the opening of a bed in another ward. |
| Abbreviations: ICU = intensive care unit; NICU = neonatal intensive care unit; A&E = Accidents & Emergency  **^a^** We treat the Antenatal and Postnatal wards as a combined Maternity ward for this analysis as nearly all antenatal patients transfer into the Postnatal ward. | |

**S1 Table. Description of 2018 QMMH inpatient wards and changes since 2012**
