# Supplementary material for: Observational study of the clinical performance of a public-private partnership national referral hospital network in Lesotho: Do improvements last over time?
Source: PLoS One. 2022 Sep 28;17(9):e0272568. doi: 10.1371/journal.pone.0272568 (PMC9518856; doi:10.1371/journal.pone.0272568)
Supplement: S2 Table — (DOCX) [file pone.0272568.s003.docx]

**S2 Table. Indicator definitions and construction at Timepoints 1 and 2**

| **Indicators** | **Disaggregation** | **Data source** | **Timepoint 1** | **Timepoint 2** | **Definition** | **Indicator Construction & Notes** |
| --- | --- | --- | --- | --- | --- | --- |
| **Capacity Indicators** | | | | | | |
| Operational beds in network | By location (wards; hospital vs. filter clinics | Administrative records | Calendar year 2012 | Calendar year 2018 | An operational bed is defined as a bed that is regularly maintained and staffed and immediately available for the care of admitted inpatients. | Hospital bed counts included 10 observational beds in the A&E Department. Mortuary beds and nursery cradles are excluded. Due to changes in calendar year 2018, operational beds were reported as an average over 12 months. On 1 January 2018, hospital beds were 425; decreasing to 347 by 31 December 2018. |
| Staff members in network | Clinical vs. non-clinical staff;  By staffing category (11) | Administrative records | Calendar year 2012 | Calendar year 2018 | Clinical staff includes physicians, dentists, nurses, nursing assistants, allied health professionals, clinical support services, radiology, pharmacy, interns, and other clinical staff. Non-clinical staff includes administrators, support staff, catering, and all others. | Includes all staff employed within QMMH-IN, either employed directly by Ts’epong or by sub-contracted organizations. |
| **Utilization Indicators** | | | | | | |
| Inpatient admissions | By location (wards; hospital vs. filter clinics) | Administrative records  (aggregate computerized patient data) | Calendar year 2012 | Calendar year 2018 | Inpatient admissions count patients in the ward where they were initially admitted. | Nursery admissions have been included for 2012 and 2018.  A&E Observation treats and observes patients who were admitted through the A&E Department while they await the opening of a bed in another ward. Of the 1290 patients admitted through the A&E Department in 2018, 648 were reassigned (admissions and relevant inpatient days) to the ward to which they were eventually transferred. Patients discharged directly from the A&E Department to home were categorized as “A&E Observation” (n=642). 2012 data did not note A&E Observation admissions, inpatient days, or deaths  Gateway Clinic did not contribute to inpatient admissions, inpatient days, or ALOS as it does not have beds and does not conduct deliveries. |
| Inpatient days | By location (ward & filter clinics) | Administrative records  (aggregate computerized patient data) | Calendar year 2012 | Calendar year 2018 | Inpatient days count the number of days patients spent in each ward. Days are attributed to the ward those days occurred in, regardless of where the patient was initially admitted. | Nursery inpatient days have been included for 2012 and 2018.  See inpatient admissions for note on A&E Observation.  Gateway Clinic did not contribute to inpatient admissions, inpatient days, or ALOS as it does not have beds and does not conduct deliveries |
| Average length of stay (ALOS) | By location (wards) | Calculated | Calendar year 2012 | Calendar year 2018 | ALOS was calculated by dividing total inpatient days by total inpatient admissions per ward. | Hospital-only ALOS is presented; filter clinic admissions and days were excluded. Wards with ALOS over 10 days were considered “long-stay wards” and were excluded from the analysis. This included the NICU for both timepoints and the Neonatal ward and Nursery for 2018 data only.  See inpatient admissions for note on A&E Observation. |
| Bed occupancy | By location (wards) | Calculated | Calendar year 2012 | Calendar year 2018 | Occupancy was calculated by dividing total number of inpatient days by the total number of operational bed days per ward . Operational bed days are the sum of days that each bed is regularly maintained and staffed and immediately available for the care of admitted inpatients over a year. | Wards sometimes had >100% occupancy because patients were admitted/transferred to a ward, but a bed was not available, so they were placed in other wards. When relevant, these individuals’ admissions and inpatient days were attributed to the ward they were intended to be placed in.  Nursery data were excluded for 2012 and 2018 as Nursery cradles are not considered operational beds.  See inpatient admissions for note on A&E Observation. |
| Ambulatory care visits | By location (hospital specialty outpatient clinics, A&E Department, Gateway Clinic, Filter clinics) | Administrative records  (aggregate computerized patient data) | Calendar year 2012 | Calendar year 2018 | Ambulatory visits were calculated as the number of unique outpatient visits to any QMMH outpatient specialty clinic (located within the hospital), the A&E Department, Gateway Clinic, and the filter clinics | This number included patients who attended the clinic in order to receive a medication refill. |
| % Accidents & Emergency (A&E) visits | None | Calculated | Calendar year 2012 | Calendar year 2018 | The percent of A&E visits was determined by dividing the number of visits to the A&E Department by the total number of ambulatory visits and multiplying by 100. |  |
| **Clinical Quality Indicators** | | | | | | |
| Stock present on crash carts | N/A | Direct observation | March 2013 | February 2020 | An inventory of the crash carts was conducted using direct observation against an established list in the A&E, Adult Surgical and Adult Medical wards. The proportion of available items was calculated against an established list. For items partially in stock, the number present was recorded. Between 69-74 items were expected on each cart depending on the ward. | A list of stock for a fully equipped crash cart was obtained from the Pharmacy Manager in 2013 and used as the denominator for both timepoints. Changes in the 2020 denominator were recorded during interviews with hospital managers. Magnesium Sulphate, Metoclopramide, and Dextrose 50% 50ml; designated as controlled substances in 2020 so are kept in locked cabinets, not on crash carts. These substances have been removed from the denominator of 2020 carts. |
| Patients triaged within 5 minutes in A&E | N/A | Direct observation | March 2013 | February 2020 | Time to triage was captured in A&E Department by a data collector sitting in A&E reception recording each patient’s arrival time and time taken into the triage room. Observations occurred in the morning, afternoon, and evening across multiple weekdays and a weekend day. | Average time to triage was calculated for Timepoint 2 observations. These data were not available for Timepoint 1. |
| **Patient Outcomes** | | | | | | |
| Hospital mortality | By location (ward);  By timing (within 24 hours of admission) | Administrative records  (aggregate computerized patient data) | Calendar year 2012 | Calendar year 2018 | Death as a percent of admissions was calculated by dividing the total number of deaths by the total number of admissions hospital-wide and by ward. The percent of deaths within 24 hours of admission was calculated by dividing the total number of deaths within 24 hours by the total number of all deaths hospital-wide and by ward. | Deaths were assigned to the ward they occurred in. A person is only admitted once to their initial ward, transfers were not included in the denominator, with minor exceptions explained below.  Overall and Neonatal/Nursery mortality figures included deaths that occurred in the Nursery in the numerator and admission of ill neonates to the Nursery in the denominator.  For 2018 data, the 148 neonates transferred from the Neonatal ward to the NICU are included in the denominator of the NICU (n=227) to calculate the mortality rate and subtracted from the denominator of the Neonatal ward (n=1,243).  See inpatient admissions for note on A&E Observation.  High mortality wards (ICU and NICU) were excluded to provide information on change in hospital mortality in all remaining wards. |
| Pediatric mortality due to pneumonia | N/A | Administrative records  (aggregate computerized patient data) | Calendar year 2012 | Calendar year 2018 | The percent of deaths in children diagnosed with pneumonia was calculated by dividing the number of deaths in children diagnosed with pneumonia by the total number of admitted children diagnosed with pneumonia and multiplying by 100. | At QMMH-IN, individuals ≤14 years of age considered pediatric patients. All individuals ≥15 years of age are considered adult patients. |
| Neonatal mortality (overall) | N/A | Administrative records  (aggregate computerized patient data) | Calendar year 2012 | Calendar year 2018 | Neonatal mortality was measured by dividing the number of neonates (≤28 days of age) who died in the NICU, Neonatal ward or Nursery by the total number of neonates admitted to those wards. | No Neonatal ward existed in QMMH in 2012. |
| NICU mortality (among very low birthweight) | Birthweight | Record review of patient charts | Random sample of 2012 charts | Random sample of 2018 charts | Birthweight for neonatal admissions was recorded on the patient chart but was not entered in the computerized patient data system. A random sample of NICU patients was selected. Charts for the selected patients were reviewed to collect birthweight and discharge status (alive at discharge or died; see indicator construction and notes).  The indicator was calculated for each timepoint by dividing the number of neonates ≤1,500g who died by the total number of neonates with birthweights of ≤1500g in the sample. | For 2012, hospital administrators provided a list of all neonates (identified by a unique hospital number) admitted to the NICU for four months (January, April, July, and October). From this list, a random sample of 100 records was drawn and charts reviewed to extract birthweight and discharge status (alive at discharge or died). Of the 127 total NICU patients for the four months in 2012, 30 (24%) charts could not be located, and 3 (2%) charts were incomplete; 77 charts were reviewed.  As discussed in the above indicator, 2018 NICU deaths were disaggregated by admission ward (NICU vs. Neonatal). For 2018, charts for 77 out of the 79 NICU direct admissions were assessed (2 charts could not be located). Additionally, a random sample of 101 of the 148 Neonatal ward admissions that were transferred to the NICU during calendar year 2018 were assessed. All 101 selected transfer charts were located and assessed. |
