# Supplementary material for: Observational study of the clinical performance of a public-private partnership national referral hospital network in Lesotho: Do improvements last over time?
Source: PLoS One. 2022 Sep 28;17(9):e0272568. doi: 10.1371/journal.pone.0272568 (PMC9518856; doi:10.1371/journal.pone.0272568)
Supplement: Attachment — Submitted filename: Response to Reviewers.pdf [file pone.0272568.s004.pdf]

Response to Reviewers:
We thank the reviewers for their comments, suggestions, and critiques. We have addressed each point they brought up below.

No	Reviewer Comment	Response
Reviewer 1		
1	This study examined a few metrics at a referral hospital in Lesotho, to ascertain if improvements seen at inception are sustained, in the context of a strained corporate relationship. It found that capacity and utilisation of the hospital increased, whilst patient outcomes improved in most areas, and quality decreased. These findings were interpreted as a promising sign of the financing model employed by this hospital, which has been, to date, poorly characterised in lower-middle income countries.<br><br>In general, this is a well-written paper. It requires a minimal language review (e.g. the abbreviation DALYs needs to be explained), and perhaps a clearer description of the aim of the paper in the abstract.	Thank you for your summary and suggestions. We have included the DALYs abbreviation and revised the study objective in the abstract.
2	The study setting requires some clarity – does QMMH function as a combined district/regional/tertiary hospital? Where does Gateway clinic refer a patient if district-level hospital services are indicated?	We have added additional information into the study setting to clarify the role of QMMH-IN within the health system.
3	The metrics selected are subject to many factors that are not really discussed, which is why I answered "No" to "Is the manuscript technically sound, and do the data support the conclusions?" Some examples inlcude a perverse incentive to increase bed utilisation rate in light of private funding, competency of individual staff/a team, competency of clinical/corporate leaders, patients' perceptions of quality, different staff	We agree that not being granted access to individual-level patient data was a major limitation of this study which is discussed in the limitations section. However, even without the individual-level data, we believe this study still makes a valid contribution to the base of academic knowledge for two main reasons.<br><br>First, to the best of our knowledge, this is the first study examining the sustained effects of a large-scale hospital PPP in a low- or middle-income country by tracking the same indicators over time. The indicators selected were appropriate at the time of the baseline assessment in

	being hired in different areas – ophthalmology beds increasing due to greater number of ophthalmologists?? etc. The PPP may have driven some of these changes, but how did it drive these? The data analysed is not granular enough to reveal this. Without acknowledgement of this, I see this study as having a questionable “valid contribution to the base of academic knowledge” (as per email from PLOS One).	2009. Because it was not initially designed as a longitudinal study and because of the evolving context of the PPP and partners, some of the changes in indicators may have been driven by multiple factors. The study design does not allow us to isolate specific drivers of change which, as the reviewer suggests, would have been ideal. However, overall, the clinical performance indicators remain better than what was observed under the government-run hospital which in and of itself is an important contribution to the community of practice and the academic literature.<br><br>Second, in early 2021, the Government of Lesotho announced its termination of the PPP contract with the private partner operating the hospital network (the Ts’epong consortium); the hospital network transitioned to government management beginning in August 2021. This will likely impact the entire health system. We believe that this study provides important context to the current controversy occurring in Lesotho and offers lessons to the broader community.
4	Quality measures are poorly selected; in addition, the number of patients assessed for time to triage is very small.	Thank you. Per the previous comment, the metrics were selected for the baseline assessment of QE II in 2009. While other quality metrics may arguably have been more appropriate, we opted to use the same measures that were initially selected in part to ensure comparability over time and ensure the utility of findings for key stakeholders including the MoH and the World Bank.<br><br>We agree that the sample of patients assessed for time to triage is small. We opted to replicate the approximate amount of time observed in previous rounds of data collection. However, due to reduced numbers of patients seen during the observation times, the overall sample was smaller than in 2012. We have added this to the study limitations section.
5	A revision of the neonatal results and discussion is required. At this stage, it has the potential to be confusing.<br><br>(a) The description of the Nursery is explained in S1 – I feel it would be	(a) We appreciate the reviewer’s feedback as we discussed this extensively during the development of this manuscript. We have included descriptions of the NICU, Neonatal ward, and Nursery into the main body of the

	wise to include this explanation in the main body of the text, so that a reader clearly understands from an early stage the differences between NICU, Neonatal ward and Nursery.<br><br>(b) Also hidden in the supplemental information (S2) is whether Nursery beds are included in the network's operational beds – please clarify in the main body of the text.<br><br>(c) I would not exclude "admissions" to nursery from the capacity/utilisation data, when deaths (i.e. outcomes that are later analysed) occur in this area.<br><br>(d) I suggest revising how neonatal mortality is calculated using neonates admitted to NICU as the denominator: what about the neonates who were admitted to the Neonatal ward and then died?<br><br>(e) Disaggregation by the admitting ward confuses things; quality of care (and outcome) is not dependent on the ward a patient was admitted.	text as recommended and hope that this gives more clarity.<br><br>(b) We excluded Nursery cradles from the operational beds for comparability because cradles were not included in the 2012 analysis. This is included in the main text under capacity measures as well as in S2 Table in greater detail.<br><br>(c) Thank you. This was not previously done in 2012. We have now added the 117 admissions direct to the Nursery for 2018 into the analysis. Additionally, we were able to access the raw 2012 data. We have opted to include the 798 admissions direct to the Nursery for 2012 into the analysis to make the data comparable. We have noted in the tables that some indicators for 2012 do not match the previously published figures due to this change. We have made these revisions to multiple tables and the main text in the results section.<br><br>(d) We now provide three separate indicators of neonatal mortality within Tables 3 and 4: (1) overall neonatal mortality, including data from the NICU, Neonatal ward, and Nursery; (2) NICU mortality; and (3) Neonatal/Nursery mortality. We believe it is important to show mortality at a ward level as we have with our other indicators. Regarding NICU mortality, if we retained only NICU admissions as the denominator while still counting all deaths that occurred in the NICU in the numerator, we do not believe this would be a fair representation of the data: 82 neonates died within the NICU in 2018; 79 were directly admitted; 148 were admitted to the Neonatal ward and then transferred to the NICU. In total, 27 direct NICU admissions deaths and 55 transfer deaths occurred in the NICU. If only deaths and admissions among neonates

		admitted directly to the NICU are considered, the NICU mortality rate for 2018 would be 35.4%. While this is very similar to the 36.1% reported in the table for all NICU deaths in 2018; we believe it is appropriate to take all deaths into account, so we have chosen to retain the 36.1%. We have added a sentence into the footnotes of Table 4 for clarification.<br><br>(e) The admitting ward could potentially impact quality of care as the Neonatal ward is not necessarily equipped to serve neonates requiring NICU services. We think it is important to show the disaggregation by admitting ward and therefore opted to disaggregate by admitting ward in Figure 1. If the reviewer and editor disagree, we're happy to revisit and consider disaggregating by birthweight alone rather than by birthweight and admitting ward.
6	Regarding statistics, I am unsure about the use of the mean square; besides being unfamiliar with it as a statistical test, and whether it was appropriately employed, I am also unsure what the results mean. The authors present the mean squares of ALoS in Table 2, but then don't really comment on what their significance.	We appreciate your feedback on this statistical test and its (lack of) ease of understanding by the reader. After further discussion with our data analyst, we have decided to remove the mean square statistic from the manuscript. As we were unable to calculate p-values due to having only aggregate data for the wards and overall, we wanted to provide the reader with a way to assess the differences between the timepoints. Providing the reader with the relative difference between the years already provides a measure of difference between the timepoints and is more easily understood by a wider audience. We believe inclusion of the mean square statistic only adds complexity without adding value, as the reviewer's comment has shown.
7	It is mentioned in lines 173-6 that "We conducted simple linear regression models with a dichotomous variable indicating the year to assess differences between ALOS for the timepoints. As we did not have access to patient-level data, p-values were not calculated. We present	Thank you for identifying this error. Originally a p-value was calculated for these measurements, but this was incorrect due to only having the average measure, not the underlying patient-level data. However, as explained above, we have also chosen to remove the mean square statistic for ALOS. We

	the mean square for each regression model.", but then contradict this in lines 211/212 "Hospital inpatient days increased by 29.0%, due to increased ALOS from 5.0 to 6.5 days (30.9%, p=0.08)". i.e. p-valued is calculated and presented.	have removed references to the mean square from Table 2 and from the methods and results sections.
8	The discussion overall is long. It does a reasonable job of condensing the large volume of data analysed, but it should be more concise, in terms of sticking to answering the research question.	We understand your concern. However, we want to ensure we adequately address the two key questions from the study protocol:<br><br>1. What is the current situation of the hospital and affiliated filter clinics in regard to availability and accessibility of services, the quantity and quality of care delivered, and patient outcomes?<br><br>2. How has availability and accessibility of services, the quantity and quality of care delivered, and patient outcomes at the QMMH-IN changed over time?<br><br>The large amount of data and complex context requires explanation. However, if the editors would like, we would be willing to address this further.
9	When it comes to discussion of the neonatal results, some of the literature quoted is not naturally comparable to the data presented.	We found that comparable neonatal literature is scarce. We believe we presented the most relevant literature we could find. For example, we cite number of US NICU bed rates to provide the reader with some reference point for the statement "33 incubators appear insufficient." We have added a caveat into the main text acknowledging that these data are not highly comparable with Lesotho. We believe it is better to include these data than presenting the results without reference to any outside literature. We have included a sentence into the limitations on the challenge of presenting our neonatal data within a wider context due to limited literature in LMICs.
10	In the Limitations section, there is too much commentary around the birthweight categories. This can simply be acknowledged.	We have made the limitations section more concise regarding birthweight categories. It now reads: "We have included neonates weighing exactly 1500 grams in the very low birthweight category to be

		consistent with 2012 data, which is slightly inconsistent with the international definition of <1500 grams.”
Reviewer 2		
1	This is a great article discussing the impact of public private partnership in a LMIC. I think it is well written with ample statistical data. I have the following comments with some minor edits.	Thank you, we appreciate your comments and suggested edits.
2	The authors chose to use crash cart supplies as a surrogate for quality, however the reason of how this relates to quality is not well discussed? Also was this an impact of lack of funding is not known. Usually low supplies can be extrapolated to higher mortality and lower quality. It would be helpful to the explain this earlier. How does a lower supplies explain a higher quality or lower mortality rate?	We have replicated the measures published for the 2012 evaluation of QMMH-IN to allow for longitudinal comparability. As reviewer 2 pointed out, there was a reduction in crash cart supplies from 86% at Timepoint 1 to 74% at Timepoint 2. While not a perfect measure of quality, we would expect that better stocked crash carts is related to lower mortality by enabling more appropriate and timely management of complications. However, with the data available, it is not possible to conclude a) what caused the change in stock, nor b) what, if any, impact it may have had on patient outcomes. Within the discussion, we posit several explanations for the observed lower mortality rates and noted the unexpected relationship between crash cart stock and the outcomes measures.
3	The data clearly shows some of the pitfalls of public private partnerships. The reason for higher ophthalmology beds with lower occupancy is certainly an outlier that does not make sense when the overall occupancy rate is higher. Was there a directive for the partnership that allots increased ophthalmology care (for e.g. a government drive to eradicate cataracts by wider screening and surgery for older patients).	To the best of our knowledge, there was no directive to increase ophthalmology care within the PPP contract, though it was a covered service. The increase in ophthalmologic beds likely resulted from the merging of the short stay medical/surgical ward, which was created between 2012 and 2018, with the ophthalmology ward in 2018. As the ophthalmology figures were also referenced by reviewer 1, we have renamed this ward as “Short Stay Medical/Surgical” to reduce confusion, so it does not appear to be only ophthalmologically focused. We have revised footnotes in the tables and the supplementary files to clarify this.<br><br>Regarding the increase in beds for this ward when the overall hospital occupancy is higher must be explained within a much larger, complex set of actions and competing interests for each actor within the PPP. We are currently developing a

		separate article to explain this complex context within more detail, but believe there is sufficient information within this current article to compare to the 2012 study.
4	The facts that this hospital is heavily directed for maternal and neonatal care should also be highlighted. Though these usually translate into higher neonatal and infant mortality, was there a difference in overall mortality reduction from cardiovascular or oncologic mortality is not known given the limited medical admissions or beds. Did this have any impact on quality?	Thank you. We clarified in the study setting that this is the highest level MCH care in the country. The very last line of Table 4 presents the change in overall hospital mortality, excluding the NICU and ICU where we would expect higher rates of mortality. We retained neonatal and nursery deaths, as those wards in theory should not have a large proportion of deaths. While we agree it's an interesting question, we don't have access to the patient level data to understand cause of death in the other wards (cardiovascular, etc), so we can only present mortality by ward as seen in Table 4. There are no oncologic services offered at this hospital; these were excluded from the PPP contract. We have expanded on excluded services in the study setting.
5	Also it would be helpful to know the scope of the public private partnership. What is the monetary impact of a per percentage drop in mortality. Did every 0.1% drop in mortality cost %100,000 or $1million is not known. What was the funding commitment or resource reimbursement per case is a data point that will help to tag a cost to the quality achieved and help benchmark future funds. Was this data available over the time period of 2012 to 2018	We agree this is a very interesting question. We will happily look into it for a future paper, if we can access the data for both 2012 and 2018.
Editor Comments		
1	Please ensure that your manuscript meets PLOS ONE's style requirements, including those for file naming.	We have corrected the file names and ensured the manuscript meets PLOS ONE's style requirements. Please let us know if there is something else that we have overlooked.
2	Please provide additional details regarding participant consent. In the ethics statement in the Methods and online submission information, please ensure that you have specified (1) whether consent was informed and (2) what type you obtained (for instance,	We have included a separate section on ethics within the methods and have addressed the following: aggregate data, anonymized data, and informed consent.

	written or verbal, and if verbal, how it was documented and witnessed). If your study included minors, state whether you obtained consent from parents or guardians. If the need for consent was waived by the ethics committee, please include this information.<br><br>If you are reporting a retrospective study of medical records or archived samples, please ensure that you have discussed whether all data were fully anonymized before you accessed them and/or whether the IRB or ethics committee waived the requirement for informed consent. If patients provided informed written consent to have data from their medical records used in research, please include this information	
3	Please include a complete copy of PLOS' questionnaire on inclusivity in global research in your revised manuscript. Our policy for research in this area aims to improve transparency in the reporting of research performed outside of researchers' own country or community. The policy applies to researchers who have travelled to a different country to conduct research, research with Indigenous populations or their lands, and research on cultural artefacts. The questionnaire can also be requested at the journal's discretion for any other submissions, even if these conditions are not met. Please find more information on the policy and a link to download a blank copy of the questionnaire here: https://journals.plos.org/plosone/s/best-practices-in-research-reporting. Please upload a completed version of your questionnaire as Supporting Information when you resubmit your manuscript.	We have included a copy of the PLOS questionnaire on inclusivity in global research with the revised manuscript. We have referenced the checklist within the main text of the article and added it as a supplementary file.

4	We note that you have stated that you will provide repository information for your data at acceptance. Should your manuscript be accepted for publication, we will hold it until you provide the relevant accession numbers or DOIs necessary to access your data. If you wish to make changes to your Data Availability statement, please describe these changes in your cover letter and we will update your Data Availability statement to reflect the information you provide.	Thank you. As this manuscript is reviewed, we will make sure to get the repository information ready for when it is accepted. It will be available at the OpenBU data repository.
