# Supplementary material for: Observational study of the clinical performance of a public-private partnership national referral hospital network in Lesotho: Do improvements last over time?
Source: PLoS One. 2022 Sep 28;17(9):e0272568. doi: 10.1371/journal.pone.0272568 (PMC9518856; doi:10.1371/journal.pone.0272568)
Supplement: Attachment — Submitted filename: Cover Letter_PlosOne_29Jun22.pdf [file pone.0272568.s005.pdf]

Boston University Center for Global Health & Development

Medical Campus
801 Massachusetts Avenue, 3rd floor
Boston, Massachusetts 02118-2605
T 617-414-1260 F 617-414-1261
www.bu.edu/cghd

BOSTON UNIVERSITY

29 Jun 2022
Editor in Chief
PLOS One

Dear Dr. Atluri:

Thank you for the opportunity to revise our research article, entitled, '*Clinical performance of a Public-Private Partnership national referral hospital network in Lesotho: Do improvements last over time?'* We are glad we sufficiently addressed most of the initial comments and appreciate the additional feedback. We have made suggested changes to the main text of the manuscript and supplementary files as appropriate. We have included a point-by-point response to the reviewers below.

Thank you for your continued consideration of our manuscript.

Sincerely,

Nancy A. Scott, DrPH, MPH
Assistant Professor of Global Health
Boston University School of Public Health

Reviewer comments and response to comments:

1. The main reason I have selected "Minor Revision" is because the results in the abstract need correcting in light of the updated results (utilisation/ALoS).
    a. **Response:** Thank you for catching this. We have updated the abstract accordingly. We have also updated the abstract and main text to reflect that the PPP has formally dissolved, seven years earlier than anticipated.

2. I would like to take the opportunity though, to suggest considering inclusion of some of your rebuttal in the Discussion - you pose very valuable points in response to my critique. The reviewer/reader may fault the metrics/indicators, but ultimately the aim of the research was to describe changes in these over time, rather than thoroughly explore or validate them. As I say, this is just a consideration... however, I do feel it would contribute to the quality of the manuscript. e.g. "The indicators selected were appropriate at the time of the baseline assessment in 2009. Because it was not initially designed as a longitudinal study and because of the evolving context of the PPP and partners, some of the changes in indicators may have been driven by multiple factors."
"...we opted to use the same measures that were initially selected in part to ensure comparability over time and ensure the utility of findings for key stakeholders including the MoH and the World Bank."
    a. **Response:** Thank you for this suggestion. We've included what you've suggested in the methods section: "The indicators selected were appropriate at the time of the baseline assessment in 2009. Because this was not initially designed as a longitudinal study and because of the evolving context of the PPP, some indicators changed over time and other measures of quality were not included. We opted to use the same measures that were initially selected to ensure comparability over time and utility for key stakeholders.
